# Limitations of Retrospective Machine Learning Models for Predicting Tracheostomy After Cardiac Surgery

**DOI:** 10.3390/diagnostics16050771

**Published:** 2026-03-04

**Authors:** Felix Wiesmueller, Johannes Rösch, Stephan Kersting, Thomas Strecker

**Affiliations:** 1 Department of Surgery, University Hospital Greifswald, University of Greifswald, 17475 Greifswald, Germany; stephan.kersting@med.uni-greifswald.de; 2 Department of Cardiac Surgery, University Hospital Erlangen, Friedrich-Alexander-University of Erlangen-Nuremberg (FAU), 91054 Erlangen, Germany; johannes.roesch@uk-erlangen.de (J.R.); thomas.strecker@uk-erlangen.de (T.S.)

**Keywords:** diagnostic validation, cardiac surgery, machine learning, deep learning, artificial intelligence, prediction model, tracheostomy

## Abstract

**Background/Objectives**: Early tracheostomy seems favorable in prolonged ventilated patients after surgery. Hence, predicting tracheostomy after cardiac surgery is essential. Recently proposed prediction models aim to support this decision-making process, but their diagnostic validity across other patient populations remains uncertain. **Methods**: A retrospective single-center study was performed at a university hospital. The patient sample included consecutive patients between 2010 and 2020 who underwent cardiac surgery. Patients who underwent tracheostomy after cardiac surgery were assigned to the intervention group. Control group patients, who had not undergone tracheostomy, were randomly assigned to the group. An existing model was evaluated by receiver operating characteristics curve analysis. Four sets of risk features were chosen depending on results from regression analysis, lasso regularization, random forest or clinical domain knowledge. Newly developed models were created using machine learning methods: random forest, naïve Bayes, nearest neighbor and deep learning. Multiple models were trained with either feature set and then assessed using confusion matrices on an independent test set. **Results**: A total of 4744 patients were included in this study. One-hundred and eighteen patients were included in the tracheostomy group. Diagnostic accuracy of the existing model showed insufficient discrimination (area under the curve (AUC) = 0.57). Likewise, newly developed models also showed overall poor diagnostic discrimination across all feature sets and algorithms. **Conclusions**: This study shows the diagnostic limitations of retrospective clinical data for the diagnostic prediction of tracheostomy, thereby informing the design of future prospective diagnostic studies. Training new models should not rely on retrospective data alone. Instead, prospective data collection and integration of physiological or imaging-based diagnostics could likely contribute to the development of a good classifier.

## 1. Introduction

Decker et al. argue that most clinical prediction models are useless in their publication about clinical risk scores [1]. The authors complain about a flood of risk scores that have surfaced over the recent years. Their observation underlines a current diagnostic problem: many prediction models lack diagnostic validity and fail to be applicable to different patient populations. In addition to classic techniques for creating prediction models, there seems to be a current substantial enthusiasm around machine learning [2]. About ten years ago, a deep neural network defeated a human professional player in the rather complex game of Go [3]. This event marked a new chapter in machine learning and was highly publicized in mainstream media. This public attention also triggered an ever growing number of medical studies that harness the power of machine learning algorithms [4].

So why bother to come up with another predictor for a seemingly rare situation such as tracheostomy after cardiac surgery?

First of all, cardiac surgery still is common with 271.5 procedures per 100,000 population per year in USA alone. It is estimated that more than 1 million cardiac surgery procedures are carried out worldwide every year [5]. Up to 10% of cardiac surgery patients need prolonged mechanical ventilation after surgery [5,6,7]. However, prolonged mechanical ventilation comes with a price: patients may get used to the ease of a ventilator and become increasingly hard to wean from it. The endotracheal tube can cause local trauma to soft tissue and is associated with higher risks of silent aspiration and pneumonia [8]. In contrast, early tracheostomy reduces trauma to oropharyngeal and laryngeal tissue, decreases airway resistance and dead space and lowers need for sedation. Consequently, patients are easier to wean from tracheostomy tubes which reduces the length of stay in intensive care units and in hospital [9]. It is unsurprising that prolonged ventilation time is associated with higher costs [10]. Early tracheostomy enables most patients to resume oral nutrition and allows early mobilization [8]. A Cochrane study showed that early tracheostomy (10 days or less after tracheal intubation) was associated with lower mortality rates [11]. Sternal wound infection is a frequently cited complication from tracheostomy. However, more recent evidence suggests that sternal wound infection may be merely associated with critically ill patients and not caused by tracheostomy [12,13]. All in all, early tracheostomy tends to be favorable in those who need prolonged mechanical ventilation after surgery. These benefits depend on timely and accurate identification of patients who will fail to wean from ventilation. This emphasizes the diagnostic relevance of early risk stratification.

With this study we aimed to (1) evaluate the diagnostic performance and external validity of an existing prediction model for tracheostomy after cardiac surgery, and (2) to assess whether machine learning-based classifiers trained on routinely available retrospective data can achieve meaningful diagnostic discrimination.

## 2. Materials and Methods

### 2.1. Aims and Criteria

The primary aim of this study was to test an existing prediction tool for tracheostomy and to explore its external validity to a patient cohort in Germany. The secondary aim was to develop new prediction models using different machine learning approaches and to validate the resulting prediction models. Inclusion criteria were cardiac surgeries from January 2010 to January 2020. Exclusion criteria were patient age younger than 18 years, heart transplantations, congenital heart surgery, endovascular procedures, death after surgery less than 48 h, tracheostomy on same day as cardiac surgery, tracheostomy more than 30 days after index procedure, and incomplete patient data.

### 2.2. Data Collection and Preparation

This retrospective study was performed at the Department of Cardiac Surgery, University Hospital Erlangen, Friedrich-Alexander-University of Erlangen-Nuremberg (FAU), Germany. It was conducted in accordance with FAU Ethics Commission guidelines and the World Medical Association Declaration of Helsinki. All patients or their legal representatives provided written informed consent for surgery and the use of their data for diagnostic and research purposes. All patient information was retrieved from an institutional database. Patient data included epidemiological parameters, follow-up data, and data from patient charts at the time of cardiac procedures, which typically include operative reports, laboratory values, monitoring data, clinical findings, and orders. Both male and female patients were included in all analysis, and sex was considered as a candidate feature during feature selection and model development. Multivariable regression analyses were performed for exploratory purposes only and were not intended to define a clinically deployable method. Continuous variables were normalized using z-scores standardization prior to model training. Data containing ordinal features were processed by dummy-coding or min-max scaling. Data were divided into training (72%), validation (8%), and test sets (20%) through stratified sampling. A separate test set consisting of all tracheostomy patients (50%) and randomly drawn patients who did not undergo tracheostomy (50%) was created for analysis of the prediction model by Wang et al. [14].

### 2.3. Evaluation of an Existing Prediction Model

Scores were calculated for each patient by adding points depending on presence of the following risk factors: renal insufficiency (2 points), emergency surgery (2 points), chronic obstructive pulmonary disease (1 point), diabetes mellitus (1 point), pulmonary edema (1 point), patient age >60 years (1 point), mixed valve and coronary artery bypass surgery (1 point), aortic surgery (1 point). Receiver operator characteristic (ROC) curve was computed with the resulting area under the curve (AUC). Confidence intervals for the AUC were obtained using non-parametric bootstrapping with 2000 resamples.

### 2.4. Selection of Putative Risk Factors

Ideal number of risk factors for later training in machine learning was determined by a random forest classifier using the attribute “feature_importances_” of the scikitLearn library “RandomForestClassifier” method. Features were analyzed for their association with postoperative tracheotomy by logistic regression using the “statsmodels” Python module (version 0.14) [15]. Four sets of eight varying risk factors were identified using regression analysis, random forest analysis, L1-regularization and clinical domain knowledge. For regression analysis, putative risk factors were first identified by univariate analysis. Features with a *p*-value of less than 0.05 were entered into multivariable analysis. Features with a *p*-value of less than 0.05 were used as a feature list for machine learning coined “Multivariate Features”. For L1-regularization, pandas and sklearn libraries were used to build a Lasso model (threshold = 0.5, penalty = ‘l1′, solver = ‘liblinear’). The resulting feature list was named ‘L1 Features’. For random forest analysis, a random forest classifier (n_estimators = 100) was created. Feature importances were calculated and sorted in descending order of importance. The top 8 features were selected to build a feature list named ‘RF Features’. For clinical domain knowledge, we picked 8 risk factors that we hypothesized to be clinically relevant to predict need of tracheostomy in patients undergoing cardiac surgery. This estimation was based on our experience as clinicians. Its features typically reflect patient morbidity or could be linked to poor respiratory status.

### 2.5. Machine Learning and Model Evaluation

The resulting feature lists were used to train four different prediction models: K-nearest neighbor, random forest, naïve Bayes, and a deep neural network (DNN). This process was repeated with every feature list. Resampling techniques tailored to the machine learning method were applied to adjust for class imbalances. For the K-nearest neighbor, the “KNeighborsClassifier” class by scikitLearn library was used. After training the model it was validated and its hyperparameter “k” (number of neighbors) was chosen based on validation accuracy. The model was then re-trained using the best “k”. Synthetic Minority Over-sampling Technique (SMOTE) was applied to training data only, using default parameters as implemented in scikitLearn library, and was not applied to validation or test sets. For classification by a random forest, a random forest classifier by the sklearn library was used. The “GaussianNB” function by sklearn library was called to create a naïve Bayes model.

The DNN consisted of a multilayer perceptron (MLP) from the sklearn library.

**Figure 1:**
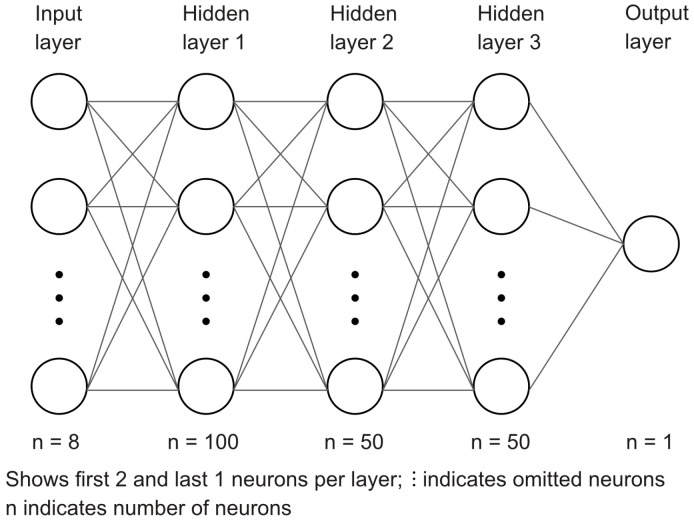
Layer architecture of the MLP classifier used in this study. The input layer consists of 8 neurons (8 features), fully connected to three hidden layers. The output layer is a single output neuron for binary classification.

The MLP consisted of an input layer corresponding to the selected features, followed by three fully connected hidden layers with 100, 50, and 50 neurons and an output layer for binary classification. The MLP architecture is illustrated in Figure 1.

Model training was performed until convergence or until predefined stopping criteria were met. For classical machine learning algorithms (random forest, naïve Bayes, K-nearest neighbor), training did not involve iterative epoch-wise optimization. For the MLP, training was performed for up to 1000 epochs with validation-based parameter tuning. Other parameters were adjusted by a grid search, such as using an adam optimizer and a constant learning rate of 1 × 10^−3^.

**Figure 2:**
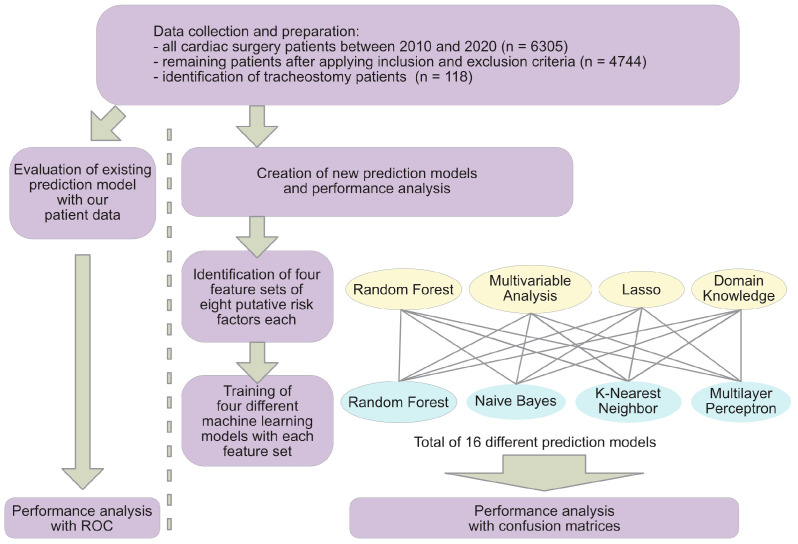
Workflow schematic summarizing cohort selection, feature engineering, model training and evaluation.

Given class imbalance, the model performance of all models was primarily assessed using confusion matrices. F1-scores can be viewed in the Supplementary Materials. A schematic of the overall workflow is displayed in Figure 2.

All data preparation and statistical analysis was done with Python 3.10 using the Juno software version 3.2, by Rational Matter Ltd., London, UK. Our approach to structuring and evaluating the machine-learning models was informed by methodological principles described in ‘Practical Deep Learning’ [16]. Workflow figure and MLP schematic were prepared using Affinity Designer software version 1.10.24, by Serif Labs Ltd., Nottinghamshire, UK.

## 3. Results

A total of 6305 consecutive patients undergoing cardiac surgery at the University Hospital Erlangen-Nuremberg between January 2010 and January 2020 were identified. One thousand five hundred and sixty-one cases were excluded due to exclusion criteria, yielding a total of 4744 valid cardiac surgery cases. Of these, 118 patients underwent tracheostomy after cardiac surgery. Clinical parameters of the cohort are summarized in Table 1.

Data were split into training, validation and test sets for the purpose of creating prediction models (Table 2).

**Figure 3:**
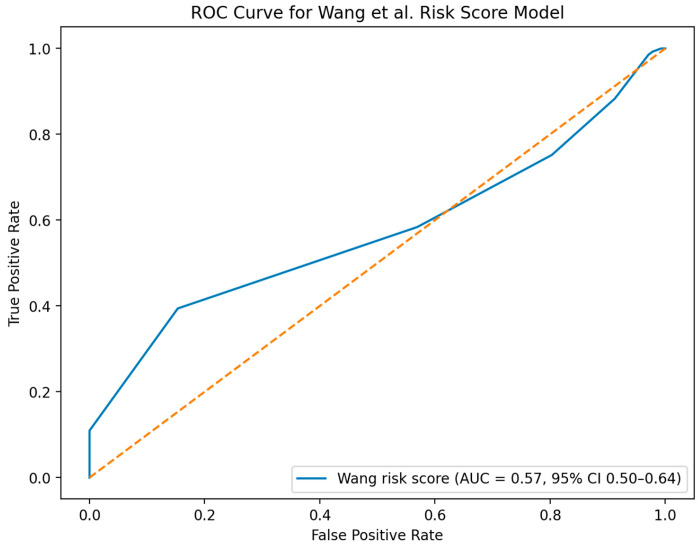
Receiver operating characteristic (ROC) curve evaluating discriminatory performance of the Wang et al. risk score with an external patient cohort [14]. Analysis was performed with complete ordinal risk score with score direction aligned to the outcome (tracheostomy). The area under the curve (AUC) was 0.57 with 95% confidence interval from 0.50 to 0.64.

Another 118 patients who did not undergo tracheostomy were randomly drawn from the cohort and combined with the tracheostomy patients to create a separate patient group to evaluate the existing prediction model by Wang et al. [14]. The ROC curve analysis (Figure 3) demonstrated near-chance discrimination.

The optimal feature count for developing a new prediction model was found to be eight factors by a random forest classifier analysis. Univariate analysis of features is summarized in Table 3. Features with a *p*-value of less than 0.05 were regarded as significant and passed on to multivariable analysis. Features with a *p*-value of less than 0.05 in multivariable analysis were added to the multivariable feature list and included pneumonia, sepsis, left ventricular ejection fraction (LVEF), intra-aortic balloon pump (IABP) runtime, patient age, ischaemia duration, coronary artery bypass graft (CABG), and gender (Table 3).

L1-regularization identified pneumonia, sepsis, LVEF, IABP runtime, CABG, gender, left ventricular assist device (LVAD) and sternal wound infection as best risk factors for classifier creation.

**Figure 4:**
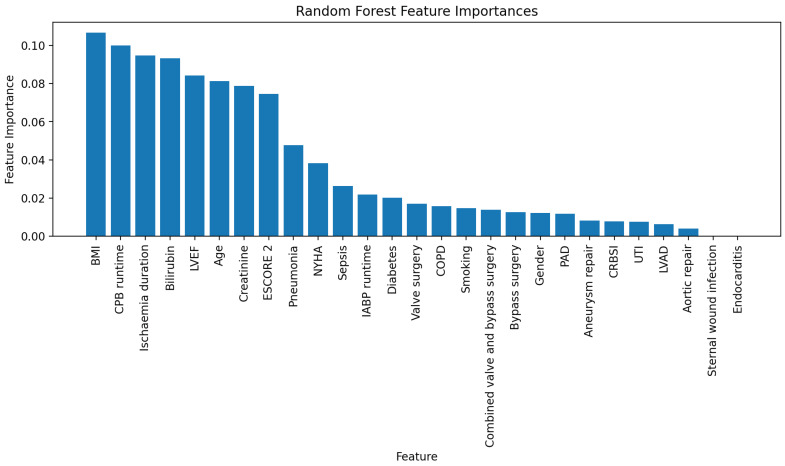
Representation of risk factor (feature) importances as determined by random forest algorithm. Features are listed in descending order of importance from left to right. BMI: body mass index; CPB: cardiopulmonary bypass; LVEF: left venticular ejection fraction; NYHA: New York Heart Association; IABP: intra-aortic balloon pump; COPD: chronic obstructive pulmonary disease; PAD: peripheral arterial disease; CRBSI: catheter-related bloodstream infection; UTI: urinary tract infection; LVAD: left-ventricular assist device.

Random forest analysis showed that body mass index (BMI), bypass duration, ischemia duration, serum bilirubin concentration, LVEF, serum creatinine concentration, patient age and European System for Cardiac Operative Risk Evaluation II (Escore-2) were ideal features (Figure 4).

**Figure 5:**
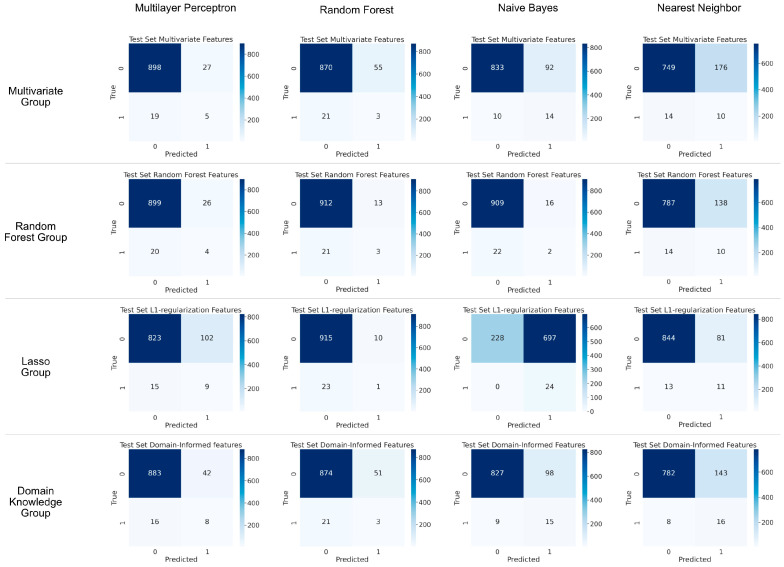
Confusion matrices illustrating performances of different prediction models with different sets of patient features on test set data. Prediction models were created using either multilayer perceptron, random forest, naïve Bayes or nearest neighbor techniques on. Features for patient feature groups were selected by either multivariate regression, random forest, L1-regularization (Lasso) or domain knowledge.

Our pick of features for clinical domain knowledge feature list included LVEF, COPD, IABP runtime, CABG, pneumonia, New York Heart Association Functional Classification (NYHA), sepsis and EScore-2. K-nearest neighbor, random forest, naïve Bayes, and a MLP classifier were built and tested with each of the four feature lists. Results were evaluated with confusion matrices (Figure 5). None of the newly developed classifiers showed satisfactory discrimination.

## 4. Discussion

In this study, neither an existing clinical risk score nor a newly developed classifier achieved meaningful discrimination for predicting tracheostomy after cardiac surgery. These findings highlight the limitations of retrospective clinical data for developing a strong model and suggest that current prediction approaches are insufficient to support reliable decision-making in real life. The present findings should not be interpreted as evidence of model generalizability, but rather as a demonstration of the limitations of retrospective data for this diagnostic task. None of the evaluated models are suitable for clinical decision-making or implementation in their current form.

Recommending elective tracheostomy following cardiac surgery is not an easy task. From our own experience with talking to patients and their families, we witnessed how many are terrified by the idea of requiring a surgically created airway. Given the circumstances, most often it is not the patients but rather their next of kin with power of attorney who have to make a decision. Clinicians, along with nurses and respiratory therapists often face challenges in determining the necessity of this procedure. A prediction model could provide guidance for clinicians, families and patients alike.

In 2022, a risk score model for predicting tracheostomy in cardiac surgery patients was published by Wang et al. [14]. Their model works by adding points depending on the presence of the following risk factors in a patient: combined valve surgery and CABG, aortic surgery, renal insufficiency, diabetes mellitus, COPD, pulmonary edema, emergency surgery, and age >60 years. A patient is categorized as having either low, intermediate or high risk to undergo tracheostomy, depending on the resulting score. Their model was developed on basis of retrospective patient data from a tertiary medical center over a time period of four years. Given the address and hospital affiliation of the authors we assume that the tertiary hospital was located in Wuhan, China. However, our analysis of their model with a typical patient cohort in Germany demonstrated near-chance discrimination (Figure 3). Possible explanation for this finding could be that patients differ between the two countries. More importantly: the need for tracheostomy was determined by a clinician in each country and not by strict clinical rules or indications. This can be explained by the fact that until this day there is no clear indication on when to proceed with elective tracheostomy. As a consequence, the dependent variable reflects clinician-dependent decision-making rather than a uniform diagnostic endpoint. If there was a clear indication, e.g., a certain lab value threshold, then intervention cohorts would be defined on the same foundation and their classifier would likely have higher external validity.

Hence, it is a valid decision to develop a separate, new prediction model from our own patient population. Random forest classifier determined that a total number of eight risk factors for model development is ideal. This number seems reasonable as prediction tools are used in a clinical setting where collecting data for an increasing number of risk factors becomes an increasingly fatiguing user experience. Nevertheless, despite careful feature selection and application of several machine learning techniques, none of the newly designed classifiers achieved sufficient meaningful discrimination (Figure 5). This consistent finding in all of our algorithms with different feature sets suggests a fundamental limitation of the available input data rather than deficiencies of modeling approaches.

There may be several reasons why our models performed poorly:

(1) Indication for elective tracheostomy was called by our clinicians. This decision-making process did not adhere to any algorithms or standards. Anecdotal evidence from our experience in intensive care suggests significant variability in the decision-making process for tracheostomy between hospitals. When talking to our in-house intensivists they reminded us that over the time period of data collection for this study there were several intensivists with different personal preferences on elective tracheostomy. Therefore, this variability introduces label noise into the dependent variable.
(2) Another factor may lie in the availability of parameters for prediction model creation. Typical parameters, such as lab values, presence of certain comorbidities, etc., may not be suitable for this particular endeavor. Intensivists usually gauge the need for tracheostomy by the progress of respirator parameters. Feedback from nurses and respiratory therapists holds valuable information, e.g., patients needing frequent airway suctioning, etc. Chest x-rays of intubated patients are acquired almost daily on most intensive care units. Simple binary diagnosis of these x-rays, such as “pneumonia” or “no pneumonia” may not be sufficient to monitor disease progression. Instead, serial and nuanced diagnostic assessments such as “worsening pneumonia” or “new infiltrates” may more accurately mirror a patient’s trajectory towards prolonged ventilation.

As a retrospective single-center study, our findings are inherently limited in generalizability and do not provide evidence for external clinical validity. In addition, the relatively small number of tracheostomy cases (n = 118) represents a substantial class imbalance, which further limits model training and predictive performance. The consistently low performance across different feature selection strategies suggests that the limitation lies not in the feature selection methodology, but in the informational content of retrospectively available clinical variables. For future studies, it would be more accurate to prospectively collect clinical and imaging data.

These data should ideally mirror the changing nature of intensive care patients meeting extubation requirements. Lee et al. published a study where they examined patients who would develop pneumonia on ICU [17]. They utilized, among other parameters, cough frequency and Mini-Mental State Examinations. These features reflect the dynamic state of a patient much more than static parameters do, e.g., prior smoking status or type of surgery. From a diagnostic perspective, tracheostomy represents an imprecise surrogate endpoint for failure to wean. Tracheostomy is influenced by institutional practice and individual clinician judgment. This represents a potential limitation shared by many published models [14,18,19]. Typically, these predictors perform well during model validation which is due to overfitting. However, with tracheostomy as the endpoint all these models fall short in their external validity and will likely fail when tested on a different cohort. Instead, patients should be dichotomized into those who failed to wean versus those who were extubated after reasonable time of prolonged mechanical ventilation. A prospective study like this should be multicentered to account for local differences in decision-making for extubation and to balance low incidence [20]. Aiming for generalizability and applicability, tested parameters should be carefully selected and advanced algorithms should be applied to generate a predictor with a low number of final input features [21,22]. Guidelines for reporting machine-learning based classifiers are available and adherence to a guideline would strengthen any publication of a new predictor [23,24,25]. Imaging data may be analyzed using convolutional neural networks (CNNs) and transformer-based architectures. CNNs could be used to monitor progress of weaning on daily chest x-rays and could pose a simple but valid method to monitor the weaning process. These types of AI-based models showed strong discriminative power when classifying radiologic imaging [26]. More specifically, detection of pneumonia on chest x-rays using CNNs has been described in the literature [27,28,29]. Combined with dynamic physiologic data, an artificial intelligence could provide clinicians with success estimates on weaning and propose tracheostomy if suited.

With the insights gained from this study, we plan to initiate a multicenter trial aimed at prospectively collecting clinical and imaging data to develop a robust prediction model with strong internal and external validity. By reframing tracheostomy prediction as a longitudinal task rather than a static binary task, future studies may overcome the shortcomings presented here. Such a model could provide clinicians, patients, and their families with a reliable tool to guide the decision-making process for tracheostomy after cardiac surgery [30]. By encouraging collaboration across institutions, we hope to standardize the approach to data collection and model development—ultimately improving outcomes for patients requiring prolonged mechanical ventilation.

## Figures and Tables

**Table 1:** Patient features of intervention (tracheostomy) and control group. Values are represented as mean ± standard derivation, and categorical variables as absolute numbers and percentages (%). Weight measured in kg, height measured in cm. All (run-)times measured in minutes. BMI: body mass index; NYHA: New York Heart Association; LVEF: left ventricular ejection fraction; PAD: peripheral arterial disease; COPD: chronic obstructive pulmonary disease; CRBSI: catheter-related bloodstream infection; UTI: urinary tract infection; IABP: intra-aortic balloon pump; CPB: cardiopulmonary bypass; LVAD: left-ventricular assist device; aneurysm repair refers to surgical repair of thoracic aortic aneurysms performed during the index cardiac procedure.

Patient Feature	Control Group (n = 4626)	Intervention Group (n = 118)	*p*-Value
Age	70.3 ± 8.7	67.0 ± 11.0	0.001
Male Gender	3459 (74.8%)	99 (83.9%)	0.031
Weight	82.9 ± 15.8	83.5 ± 21.8	0.752
Height	172.5 ± 8.5	172.3 ± 25.9	0.929
BMI	24.0 ± 4.3	24.2 ± 4.6	0.575
NYHA	1: 1961 (42.4%)|2: 979 (21.2%)|3: 1305 (28.2%)|4: 381 (8.2%)	1: 35 (29.7%)|2: 16 (13.6%)|3: 39 (33.1%)|4: 28 (23.7%)	<0.001
LVEF	44.5 ± 17.6	61.5 ± 23.4	<0.001
Creatinine	1.4 ± 0.9	1.1 ± 0.8	0.002
Bilirubin	1.1 ± 0.8	0.7 ± 1.1	<0.001
ESCORE 2	9.7 ± 4.0	6.8 ± 3.7	<0.001
Diabetes	1437 (31.1%)	46 (38.9%)	0.083
PAD	454 (9.8%)	14 (11.9%)	0.561
COPD	439 (9.5%)	25 (21.2%)	<0.001
Smoking	858 (18.6%)	25 (21.2%)	0.544
Sternal wound infection	66 (1.4%)	0 (0.0%)	0.364
CRBSI	45 (1.0%)	5 (4.2%)	0.003
Endocarditis	17 (0.4%)	0 (0.0%)	1
UTI	136 (2.9%)	6 (5.1%)	0.282
IABP runtime	36.0 ± 73.3	9.2 ± 31.6	<0.001
CPB runtime	112.9 ± 51.8	94.6 ± 126.7	0.117
Ischemia time	63.9 ± 34.6	53.8 ± 38.4	0.005
Bypass surgery	2559 (55.3%)	41 (34.8%)	<0.001
Valve surgery	977 (21.1%)	32 (27.1%)	0.145
LVAD	126 (2.7%)	6 (5.1%)	0.209
Combined valve and bypass surgery	632 (13.7%)	29 (24.6%)	0.001
Aneurysm repair	156 (3.4%)	6 (5.1%)	0.45
Pneumonia	257 (5.6%)	50 (42.4%)	<0.001
Sepsis	243 (5.3%)	31 (26.3%)	<0.001
Aortic repair	163 (3.5%)	4 (3.4%)	1

**Table 2:** Number of patients divided among subsets to train models (Training), to validate during training (Validation) and to evaluate final models (Test). Values are presented as number (percentage). Percentages are calculated within each subset to reflect the stratified sampling procedure.

	Training Set	Validation Set	Test Set
No Tracheostomy (n)	3238 (97.5%)	463 (97.5%)	925 (97.5%)
Tracheostomy (n)	82 (2.5%)	12 (2.5%)	24 (2.5%)

**Table 3:** (**a**)**.** Univariate analysis of risk factors (features) for tracheostomy in cardiac surgery patients. BMI: body mass index; NYHA: New York Heart Association; LVEF: left ventricular ejection fraction; PAD: peripheral arterial disease; COPD: chronic obstructive pulmonary disease; CRBSI: catheter-related bloodstream infection; UTI: urinary tract infection; IABP: intra-aortic balloon pump; CPB: cardiopulmonary bypass; LVAD: left-ventricular assist device. (**b**). Multivariable regression analysis of risk factors (features) for tracheostomy after cardiac surgery.

(**a**)			
**Patient Feature**	**Coefficient**	**Standard Error**	***p*** **-Value**
Pneumonia	2.53	0.20	<0.001
Sepsis	1.86	0.22	<0.001
ESCORE 2	0.18	0.02	<0.001
LVEF	−0.81	0.11	<0.001
IABP runtime	1.42	0.20	<0.001
NYHA	1.33	0.27	<0.001
Bypass surgery	−0.84	0.20	<0.001
COPD	0.94	0.23	<0.001
Combined valve and bypass surgery	0.72	0.22	0.001
Age	0.34	0.11	0.001
CRBSI	1.51	0.48	0.002
Creatinine	0.16	0.05	0.003
Ischemia duration	0.12	0.05	0.011
Gender	0.56	0.25	0.026
Bilirubin	0.08	0.04	0.031
Diabetes	0.35	0.19	0.068
Valve surgery	0.33	0.21	0.117
LVAD	0.65	0.43	0.13
UTI	0.57	0.43	0.183
CPB runtime	0.04	0.04	0.219
Aneurysm repair	0.43	0.43	0.315
PAD	0.21	0.29	0.462
Smoking	0.17	0.23	0.467
BMI	−0.05	0.09	0.575
Aortic repair	−0.04	0.52	0.938
Endocarditis	−17.11	7865.56	0.998
Sternal wound infection	−26.73	487,730.37	1
(**b**)			
**Patient Feature**	**Log Odds Ratio**	**Standard Error**	***p*** **-Value**
Pneumonia	1.91	0.22	<0.001
Sepsis	1.31	0.25	<0.001
LVEF	−0.56	0.12	<0.001
IABP runtime	0.81	0.24	0.001
Age	0.36	0.13	0.006
Ischemia duration	0.14	0.05	0.009
Bypass surgery	−0.57	0.26	0.025
Gender	0.60	0.28	0.029
COPD	0.42	0.26	0.104
Bilirubin	0.06	0.04	0.111
ESCORE 2	0.05	0.03	0.119
CRBSI	0.51	0.52	0.328
Combined valve and bypass surgery	−0.11	0.28	0.682
Creatinine	0.03	0.09	0.776
NYHA	0.05	0.30	0.860

## Data Availability

Dataset available on request from the authors.

## Supplementary Materials

The following supporting information can be downloaded at: https://www.mdpi.com/article/10.3390/diagnostics16050771/s1.

## Abbreviations

The following abbreviations are used in this manuscript:

AI	Artificial intelligence
AUC	Area under curve
FAU	Friedrich-Alexander-University Erlangen-Nuremberg
ROC	Receiver operator characteristic
DNN	Deep neural network
SMOTE	Synthetic minority over-sampling technique
MLP	Multilayer perceptron
BMI	Body mass index
NYHA	New York Heart Association
LVEF	Left ventricular ejection fraction
PAD	Peripheral arterial disease
COPD	Chronic obstructive pulmonary disease
CRBSI	Catheter-related bloodstream infection
UTI	Urinary tract infection
IABP	Intra-aortic balloon pump
CPB	Cardiopulmonary bypass
LVAD	Left-ventricular assist device
CNN	Convolutional neural network
