# Supplementary material for: Limitations of Retrospective Machine Learning Models for Predicting Tracheostomy After Cardiac Surgery
Source: Diagnostics (Basel). 2026 Mar 4;16(5):771. doi: 10.3390/diagnostics16050771 (PMC12984356; doi:10.3390/diagnostics16050771)
Supplement: Supplementary file 1 [file diagnostics-16-00771-s001.zip › F1-scores.pdf]

**Model name**	**F1 (class 0)**	**F1 (class1)**
MLP with multivariate group features	0.98	0.18
MLP with random forest group features	0.98	0.15
MLP with lasso group features	0.93	0.13
MLP with domain knowledge features	0.97	0.22
Random Forest with multivariate group features	0.96	0.07
Random Forest with random forest group features	0.98	0.15
Random Forest with lasso group features	0.98	0.06
Random Forest with domain knowledge features	0.96	0.08
Naive Bayes with multivariate group features	0.94	0.22
Naive Bayes with random forest group features	0.98	0.10
Naive Bayes with lasso group features	0.40	0.06
Naive Bayes with domain knowledge features	0.94	0.22
Nearest Neighbor with multivariate group features	0.90	0.09
Nearest Neighbor with random forest group features	0.96	0.13
Nearest Neighbor with lasso group features	0.96	0.13
Nearest Neighbor with domain knowledge features	0.91	0.07
